# Improved phenotyping procedure for evaluating resistance in rice against gall midge (*Orseolia oryzae*, Wood-Mason)

**DOI:** 10.1186/s13007-021-00823-5

**Published:** 2021-11-29

**Authors:** Ling Cheng, Fugang Huang, Zhe Jiang, Baiyi Lu, Xiaohui Zhong, Yongfu Qiu

**Affiliations:** 1 Agricultural College, Guangxi University, Nanning, 530005 Guangxi China; 2 College of Agriculture, Yangtze University, Jingzhou, 434025 Hubei China

**Keywords:** Rice gall midge (RGM), Chewing insect, High-throughput phenotyping method (HTPM), Gene mapping

## Abstract

**Background:**

The rice gall midge (RGM, *Orseolia oryzae*, Wood-Mason), an important stem-feeding pest worldwide, has caused serious production losses over the past decades. Rice production practices indicate that the most reliable method for managing RGM is the deployment of cultivars that incorporate host resistance. However, the conventional phenotypic screening method of rice resistance to RGM suggested by the International Rice Research Institute (IRRI) has been used for approximately 30 years, and only 12 rice varieties/lines (including controls) can be evaluated in one tray. It is not suitable for high-throughput phenotyping of rice germplasm. Moreover, a suitable method to prepare samples for molecular biological studies of rice resistance against RGM is imperative with the rapid development of modern molecular techniques.

**Results:**

The proper density of seedlings/RGM was determined for four seeding arrangements. A high-throughput phenotyping method (HTPM) for 60 lines/varieties infested with 36 female RGM adults in one tray, as described by method 4–3 (seeded 60 lines/varieties), was developed and verified using mutant screening. Furthermore, one RGM resistance gene flanked by markers 12RM28346 and 12RM28739 on chromosome 12 was simultaneously detected using method 2–2 (seeded 30 lines/varieties in one tray) treated with 24 RGM and analyzed using conventional and simplified grading systems. Genetic analysis of the RGM resistance gene was confirmed using a method identical to that suggested by IRRI. Finally, one bucket with 24 seedlings treated with at least five female RGM adults was efficacious and could offer adequate samples for insect development observation or molecular biological studies.

**Conclusion:**

A highly efficient and reliable procedure for evaluation of resistance in rice to RGM was developed and improved, and was verified through mutant screening, gene mapping, genetic analysis, and insect growth and development observations.

## Introduction

Rice (*Oryza sativa* L.), one of the most important food crops, supplies more than 20% of the global dietary energy and feeds more than half of the world’s population [1]. Owing to a fast-growing population, global rice consumption is projected to increase from 450 million tons in 2011 to approximately 490 million tons in 2020, and 40% more rice production is needed by 2050 to meet people’s demand for food [2, 3]. However, many adverse factors, such as diseases and pests, pose a serious threat to rice production. Among these threats, the rice gall midge (RGM, *Orseolia oryzae*) is considered to be the third most destructive pest after borers and planthoppers [4].

The RGM chews the rice stem tip and forms a gall that can cause production losses. To complete the invasion, hatched larvae crawl on the rice leaves with the help of a drop of water and invade the shoot from the gap between the leaf and sheath or edge of the tongue, and then begin to chew the leaf sheath. The rice growing point can be damaged, and the leaf sheath elongates into a gall that appears as a silver shoot or multiple tillers. Therefore, the percentage of silver shoots (PSS) is the most important and obvious indicator of resistance evaluation after infestation. Seedlings with galls are also called onion emergence in Chinese, as the gall is similar to an onion. Furthermore, seedlings with inflated shoots or multiple tillers are also considered to be susceptible according to the Standard Evaluation System of the RGM described by the International Rice Research Institute (IRRI) [5–7].

It is a chewing pest prevalent in rice-growing areas of Asia, such as India, Bangladesh, Sri Lanka, and Southern China. An estimated annual yield loss of $550 million has been reported in Asia [8]. Practical experience suggests that host plant resistance is the most effective and economical method for pest management, including RGM and planthoppers. Therefore, exploring resistance germplasm and genes and incorporating them into rice varieties is a necessary and important means of managing insect pests.

Less progress has been achieved in the study of host plant resistance to RGM in the past decades than to rice diseases or planthoppers. For instance, only 12 RGM resistance genes have been detected, of which nine (*Gm1*, *Gm2*, *Gm4*, *Gm5*, *Gm6*, *Gm7*, *Gm8*, and *Gm11*) have been mapped onto rice chromosomes [9], whereas 38 brown planthopper (BPH) resistance genes have been mapped in detail and nine genes have been cloned [10, 11]. This lack of RGM resistance genes detection restricts their use in marker-assisted selection in rice breeding programs. The most likely reason is that RGM was considered a type of secondary rice pest and valued less worldwide [12]. An increased focus and studies on RGM as a serious crop hazard would help the development of modern molecular biological techniques to address this lacuna. Moreover, the rice-RGM interaction is an important example for studying plant-chewing insects. Therefore, it is important to accelerate studies on host plant resistance to RGM. However, the conventional evaluation method described by IRRI has been applied for 30 years, and to meet the requirements of our study using this method was difficult. For instance, with the development of high-throughput sequencing technologies, a large amount of plant genotype data can be easily obtained; however, the corresponding accurate phenotype data is difficult to complete because only ten lines can be evaluated in a single tray [6]. Moreover, the method is not compatible with modern molecular studies, as it is not accurate enough. Resistance evaluation of one or two rice lines is needed to study the interaction of rice-GM. Generally, accurate phenotype data are typically required in the studies of resistance material identification, mutation screening, gene/quantitative trait locus (QTL)-mapping and cloning, or resistance mechanism characterization. Therefore, developing a high-throughput phenotyping method (HTPM) and improved evaluation accuracy is one of the most important and imperative requirements for promoting studies on host plant resistance against RGM.

The purpose of this study was to (i) determine the exact density of seedlings/RGM for different scientific experiments, (ii) develop and improve the phenotyping procedure for rice resistance against RGM at the seedling stage; and (iii) verify the effect and efficiency of the developed or improved phenotyping procedure. This would be beneficial for rice GM resistance breeding programs and molecular biological studies of rice-GM interactions.

## Material and methods

### Plant materials

The varieties Kangwenqingzhan (abbreviated KW) and ARC5984 are highly resistant to RGM insects, whereas rice line 9311 is susceptible to RGM. KW and ARC5984 have been detected to carry the RGM resistance genes *Gm6* and *Gm5*, respectively [9, 13]. Backcrossing generations and near-isogenic lines (NILs) with a 9311 genetic background were developed according to the method described by Li et al. [9] and Zhou et al. [13]. Specifically, marker YW21 was applied to detect the heterozygosity at the *Gm6* region of BC_2_F_1_ individuals, which were self-pollinated to develop the BC_2_F_2_ population.

CL6 is a newly detected RGM resistance variety that was crossed with 9311 to develop F_1_ individuals. The positive F_1_ individuals were self-pollinated twice to develop the F_3_ population, which was applied to map the RGM resistance gene.

### Mutant development

To develop susceptible lines of KW, it was mutagenized by ^60^Co-γ ray irradiation with an intensity of 5.5 Gy/min for 1 h at the Zhejiang Academy of Agricultural Sciences. Approximately 10,000 treated M0 individuals were self-pollinated and 9,000 M1 lines were obtained in the spring of 2017, and five individuals of each M1 line were self-crossed to develop M2 lines in the autumn of 2017 and spring of 2018. Finally, a total of 21,000 M2 lines were surveyed with RGM from 2017 to 2019.

### RGM insects

Silver shoots with RGM insects were collected from rice paddies in Nanning, Guangxi Province and cultivated in a bucket (25 cm diameter, 18 cm height) with 15 cm height paddy in light-transmitting nylon cages (50 × 50 × 100 cm in length, width, and height, respectively) at Guangxi University. The emerged adults were collected and reared on the seedlings of susceptible line 9311, which was covered by a nylon dome (44 × 34 × 44 cm in length, width, and height, respectively) with a black cotton cloth (1 × 1 m in length and width, respectively) to maintain a dark environment, and water was sprayed overhead until a small drop of water could be observed on the surface of leaves to maintain moisture overnight during the inoculation period. Water spraying was repeated every morning and dusk and continued for 2 d, after which the cloth and dome were removed. The seedlings were managed as per standard cultivation practices, and female adult insects were collected and used 20–25 d after infection (DAI).

### Facilities for RGM resistance test

An insecticide-free and pest-exclusion-net-room with natural light (30 × 25 × 12 m in length, width, and height, respectively) was used to test RGM resistance. For seedling growth, seedling nursery ferrous or plastic trays (58 × 38 × 9 cm in length, width, and height, respectively), plastic buckets, and a light-transmitting nylon dome were used. It is worth noting that the damage caused by mice, rice planthoppers, ants, and spiders during the entire insect resistance evaluation process should be considered. The light-transmitting nylon dome can restrict mice, rice planthoppers, and spiders; and ants can be prevented using the water insulation method.

### Procedures for the RGM resistance test

To perform the RGM resistance test, the field paddy was completely soaked and mixed, and foreign substances such as stone and rice straws were removed before use. The paddy was then packed in a tray, bucket, or cup at approximately three-quarters of the volume.

Full rice seeds packed with nylon bag (5 × 7 cm in length and width, respectively) were soaked in a bucket with enough water and placed in an incubator at 37 °C in the dark. The soaked seeds were washed once or twice until germinated (approximately 36–48 h) and germination was accelerated by maintaining in a moist environment for 24–36 h. Seeds with 1 cm buds can be seeded in a tray, bucket, or cup according to the test requirements. All the seeded trays, buckets, or cups were moved into a green room at 30 °C and covered with equipment of the same size to maintain a dark environment for 3 d. Finally, the 7-d-old seedlings (approximately two leaves) were infested with a specific number of female RGM adults according to the experimental design, and the infested seedlings were treated as described above. The PSS of each line was recorded at 20–25 DAI.

**Fig. 1:**
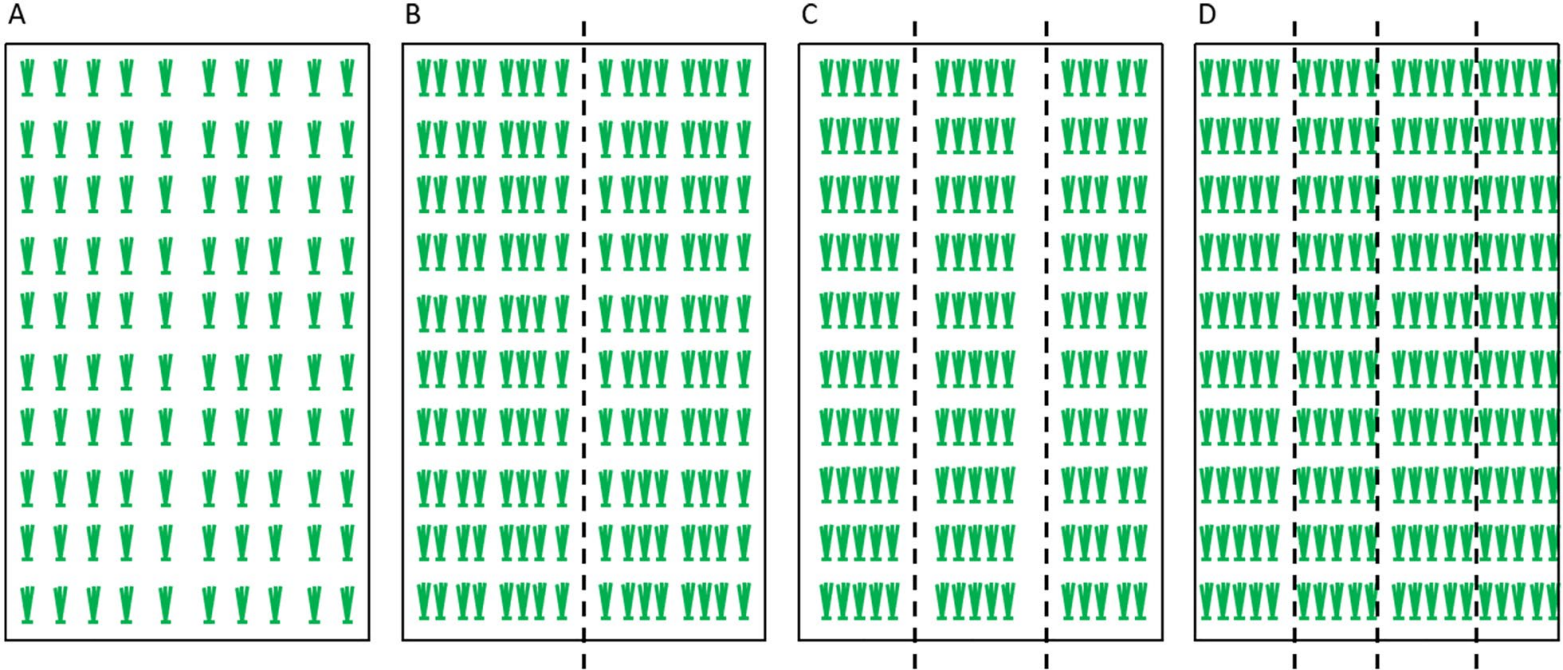
Seeding arrangements for rice gall midge (RGM) free-feeding test. One tray (58 × 38 × 9 cm in length, width, and height, respectively) was divided into 1 (**A**), 2 (**B**), 3 (**C**), or 4 (**D**) equal blocks lengthwise, and named as methods 1, 2, 3, and 4, respectively. The block was divided into 12 plots, where 12 lines, including one or two controls and 20 seedlings of each line, were arranged for method 1. Fifteen plots with a total of 16, 10, and 10 seedlings planted equidistant in each plot were set up for methods 2, 3, or 4, respectively

To conduct an insect-free choice test (in which insects are free to infest the different rice varieties/lines offered), one tray was divided into 1 to 4 equal blocks lengthwise that were labeled as methods 1 to 4, respectively (Fig. 1). The block was divided into 12 plots, where 12 lines, including one or two controls and 20 seedlings of each line, were arranged for method 1. Fifteen plots with a total of 16, 10, and 10 seedlings planted equidistant in each plot were set up for methods 2, 3, or 4, respectively. For the RGM resistance test of the F_3_ or BC_2_F_3_ lines, one line each of 9311 (susceptible control) and KW (resistance control) was used for each tray. Two or three replicates of all treatments were performed.

To determine the appropriate number of insects for the no-free-choice test (in which insects can only infest one rice variety/line), each bucket with 24 seven-day-old seedlings was infested with 1 to 6 RGM insects and covered with a single light transmitting nylon net (28 cm diameter, 80 cm height). The PSS of each bucket was recorded at 20–25 DAI. Three buckets were designed for each treatment, and two replicates of the test were performed.

### Indicators and criteria for RGM resistance evaluation

The damage level of rice plants against RGM was divided into six grades as described by IRRI: 0, free of damage; < 5%, highly resistant; 6%–10%, resistant; 11%–20%, moderately resistant; 21%–50% susceptible; and > 50%, highly susceptible (Table 1). Seedlings with inflated shoots or multiple tillers were considered resistant as the larvae could not damage the stem tip continuously and finally died, according to observations from our previous studies [9, 13]. Therefore, the evaluation criterion was simplified to four grades as follows: < 5%, highly resistant; 6–15%, resistant; 16–50%, susceptible; and > 50%, highly susceptible (Table 1). A test was considered valid only when the PSS of the susceptible control group was ≥ 60% [6, 7].

**Table 1:** Rice gall midge (RGM) resistance evaluation criterion

Indicator	Evaluation grade					
Reference grade						
TSSR	Free of damage	< 5%	6–10%	11–20%	21–50%	> 50%
Score	0	1	3	5	7	9
Rank*	FOD	HR	R	MR	S	HS
Improved grade						
TSSR	< 5%		6–15%		16–50%	> 50%
Rank	HR		R		S	HS

*FOD, HR, R, MR, S, and HS indicate free of damage, high resistance, resistance, middle resistance, susceptibility, and high susceptibility, respectively

Each seedling was evaluated, and the PSS of each line was recorded after infestation when the susceptible control showed fully exerted galls at 20–25 DAI. The PSS of each line was averaged in one tray/bucket, and was scored as an average of the replicated tests for the final PSS value. A higher PSS indicates a greater susceptibility.

### Experiments designed for specific study requirements

The RGM insects are generally applied without the free-feeding treatment to detect larval developmental characteristics, determine gene expression by quantitative reverse transcription-PCR (qRT-PCR), or study the transcriptome and metabolome. Therefore, no-free-feeding test methods were needed to meet the specific requirements of this study. Specifically, one bucket with 24 seven-d-old seedlings each of NILs and 9311 were treated with six female RGM adults infested for 0, 1, 3, and 4 d. Insect growth and development in seedlings with the contrasting genotypes were observed as described by Li et al. [9]. The qRT-PCR was conducted with total RNA using the *PrimeScript™* RT Reagent Kit with gDNA Eraser (Perfect Real Time) (TaKaRa) according to the manufacturer’s instructions according to the manufacturer’s instructions as described by Zhou et al. [13].

### Gene mapping

No RGM resistance gene has been previously reported in CL6, and one population including 116 F_3_ lines was developed to verify the effect of the improved method in resistance gene mapping. Specifically, all F_3_ lines were surveyed using RGM according to method 2–2 (Fig. 1B). Fifteen 16-seedling lines were seeded in one plot and two blocks were arranged in one tray. One line each of 9311 and KW was randomly seeded among the tested lines. A total of 24 female RGM adults were treated in one tray. The bulked segregate analysis method was applied to detect the linked markers associated with the resistance gene based on the phenotype of the F_3_ population [14]. Ten extremely resistant or susceptible lines constituted two DNA pools that were surveyed using simple-sequence repeats and insertion-deletion markers from 12 rice chromosomes. The polymorphic markers between DNA pools, together with markers around the region of interest, were surveyed to detect genotypes of the F_3_ population. A local genetic map was developed using IciMapping 4.0 [15]. A permutation test (1000 times) was used to calculate the logarithm of odds (LOD) threshold, and QTL IciMapping interval mapping was used to analyze the genetic linkage maps and map population phenotypes to identify associated resistance genes or QTLs.

### Statistical analysis

Data were analyzed using one-way ANOVA, and means were compared using the least significant difference test. The PSS (%) was arcsine-transformed before analysis. The least significant difference test and correlation analysis were performed using SPSS (version 13.0; SPSS Institute Inc., Chicago, IL, USA).

## Results

### Determination of density of seedlings/RGM for the free-feeding resistance evaluation

**Fig. 2:**
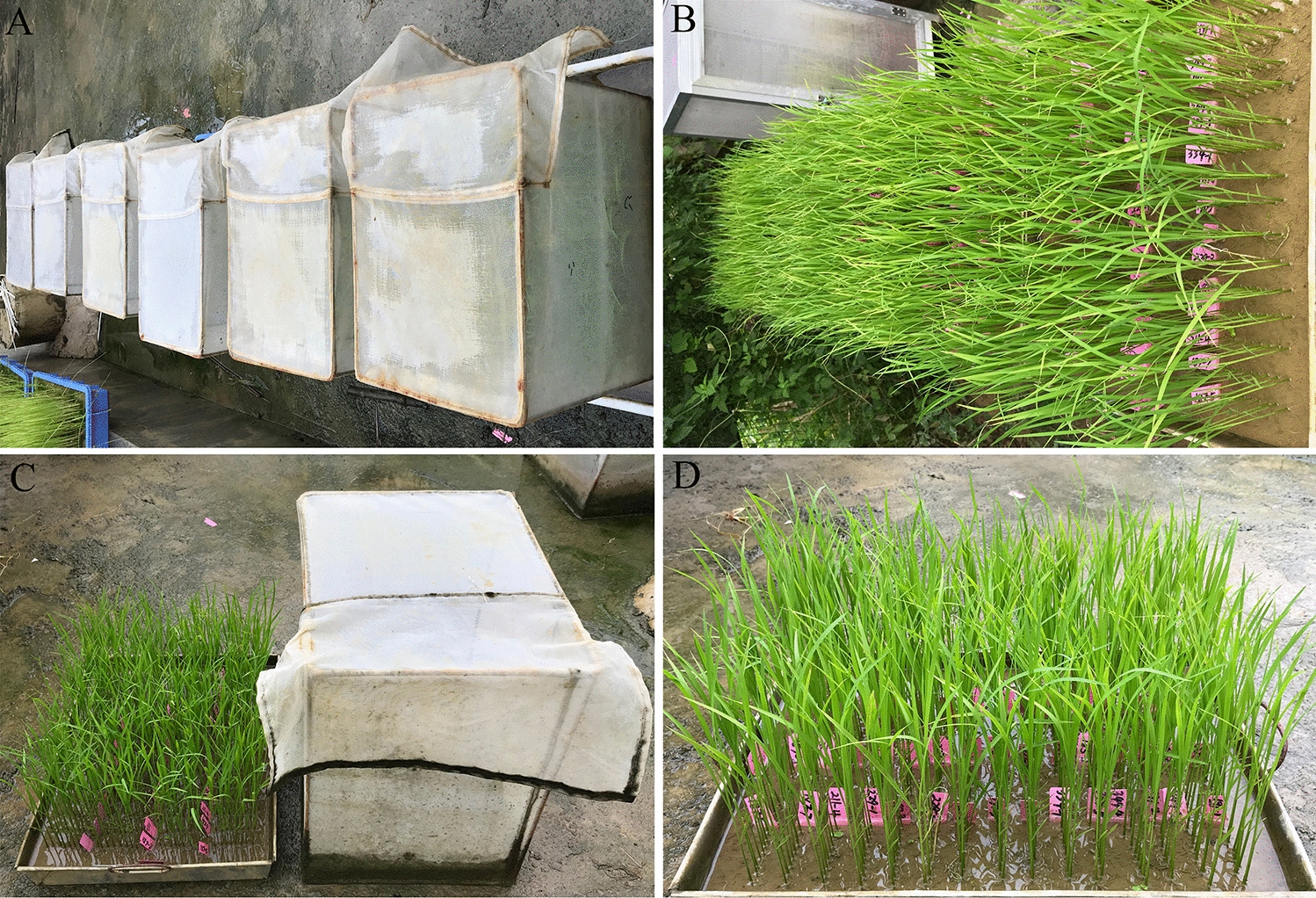
Overview of high throughput phenotyping method (HTPM) of rice against rice gall midge (RGM) at the seedling stage. **A** Seedlings treated with RGM at one day after infestation (DAI); **B**–**D** Seedlings treated with RGM at 7 DAI

A significant negative correlation was observed between PSS and density of seedlings/RGM (*r* = 0.904, *p* < 0.01). The PSS ranged from 64.2% to 90.8% when the density of seedlings/RGM varied from 13 to 19, and it was > 90% or < 60% when the density of seedlings/RGM was ≤ 10 or > 20, respectively (Table 2). According to the criterion described by the IRRI, the PSS of the control was ≥ 60% and considered to be efficient. The most efficient treatment with the largest number of seedlings and fewer insects was method 4–2, which contained 60 plots infected with 24 RGM (82.2%). The least number of RGM insects needed for the treatment was 12 plots with 12 RGM (Table 2; Figs. 1 and 2).

**Table 2:** Insect density (seedlings/RGM) determination for free feeding test

Method	Number of block	Number of plots	Number of RGMs	Number of seedlings/RGM	PSS ± SE
1	1	12	12	19	86.5 ± 1.8
2–1	2	30	12	29	40.1 ± 5.8
2–2	2	30	24	15	90.8 ± 2.3
3–1	3	45	12	30	58.1 ± 10.8
3–2	3	45	24	13	74.9 ± 4.2
3–3	3	45	36	10	91.5 ± 1.7
4–1	4	60	12	35	38.1 ± 11.8
4–2	4	60	24	14	82.2 ± 6.4
4–3	4	60	36	10	93.6 ± 1.5
4–4	4	60	48	9	99.1 ± 3.6

### Determination of density of seedlings/RGM for the no-choice feeding resistance evaluation

The PSS was less than 60% when the buckets were treated with 1–4 insects. However, it was 80.4% and 97.0% when seedlings were infested with 5 or 6 RGM, respectively (Table 3). The result suggested that at least five female RGM were needed for this type of test.

**Table 3:** Insect density (seedlings/RGM) determination for no choice feeding test

Number of RGM	PSS ± SE	Number of seedlings/RGM ± SE
1	7.4 ± 3.0	20 ± 1
2	44.4 ± 12.9	10 ± 2
3	42.2 ± 7.4	6
4	39.2 ± 11.5	5
5	97.0 ± 5.2	5
6	80.4 ± 11.4	4

### Verification of the improved indicators or procedure

#### ^60^Co-γ ray mutant screening using HTPM

To test the efficiency of HTPM for rice GM resistance evaluation, the improved method 4–3 in which 36 RGM were used for seedlings infestation was evaluated with the ^60^Co-γ ray mutagenized population of KW for susceptibility to RGM. Specifically, 15 lines, including one line of the susceptible 9311, were randomly seeded in each plot, and a total of four blocks were arranged in one tray, and 36 female adult RGM were infested. Sixty lines, including controls, could be evaluated in one tray, and 30 to 50 trays were arranged for a single test according to the number of RGM (Fig. 2). Therefore, 1800 to 3000 tested lines could be evaluated simultaneously. Subsequently, 21,000 M2 lines were surveyed using this method, and two susceptible lines were identified.

#### Genetic analysis of the ***Gm6*** gene using the BC_2_F_2_ (9311/KW) population

**Fig. 3:**
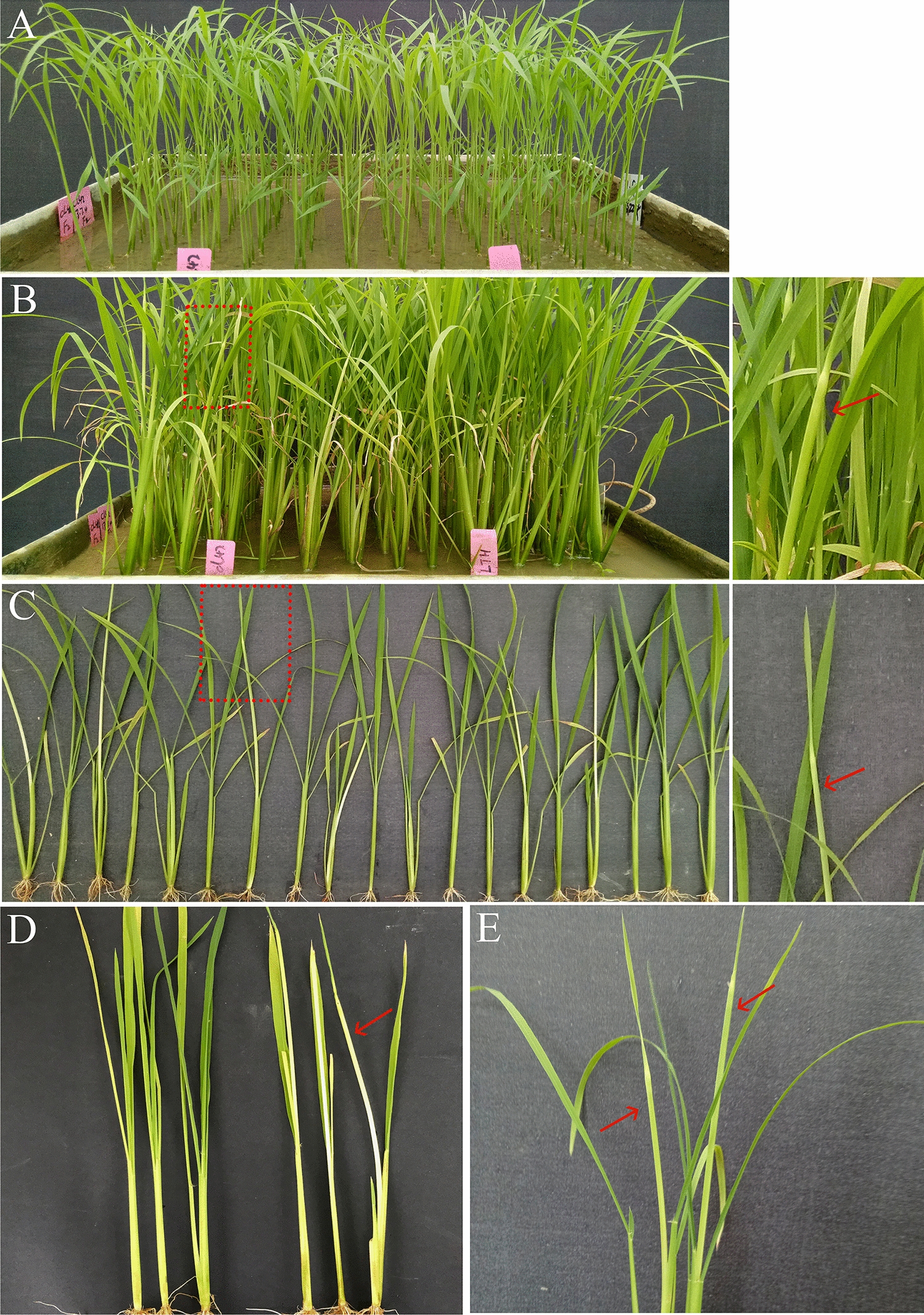
Conventional phenotyping procedure of rice against rice gall midge (RGM) at seedling stage. **A** 5 days after RGM infestation; **B** 25 days after RGM infestation; **C** seedlings of one line at 25 days after infestation (DAI); **D** resistant seedlings (left), susceptible seedlings (right); **E** individuals with galls. Red arrows indicate rice galls

To test method 1, as described in Fig. 1A, genetic analysis of the BC_2_F_2_ population carrying the known RGM resistance gene *Gm6* was conducted. Ten tested lines were seeded on average according to method 1, with one line each of KW and 9311 randomly seeded in one tray (Figs. 1 and 3). Approximately 400–500 seedlings of the BC_2_F_2_ population were surveyed with RGM, and a total of ten populations were tested. Consequently, the ratio of susceptible and resistant seedlings of each selected BC_2_F_2_ population varied from 0.27 to 0.39, and all of them abided by the 3:1 distribution according to the chi-square test (Table 4). A total of 1078 seedlings were detected with silver shoots with gall and evaluated as susceptible, and 3364 seedlings were recorded as resistant. The ratio of resistant and susceptible seedlings was also in accordance with a 3:1 distribution (*χ*_*c*_^2^ = 0.95 < *χ*_*c*_^2^_0.05,1_ = 3.84). The results suggested that one dominant resistance gene controls RGM resistance in the developed population, and the method could be used effectively to conduct genetic analysis of certain RGM resistance genes using population mapping.

**Table 4:** Genetic analysis of *Gm6* gene using BC_2_F_2_ (9311/KW) population

BC_2_F_2_ line	Number of susceptible plants	Number of resistant plants	Total number	R: S*	*χ*_*c*_^2^
L1	103	355	458	0.29	1.53
L2	122	379	501	0.32	0.11
L3	106	357	463	0.30	1.09
L4	108	310	418	0.35	0.15
L5	109	343	452	0.32	0.18
L6	135	346	481	0.39	2.41
L7	98	325	423	0.30	0.75
L8	90	325	415	0.28	2.42
L9	121	301	422	0.40	3.03
L10	86	323	409	0.27	3.44
Total	1078	3364	4442	0.32	0.95

^*^R, resistance, S, susceptible

#### Identifying the RGM resistance gene with the simplified indicators

**Fig. 4:**
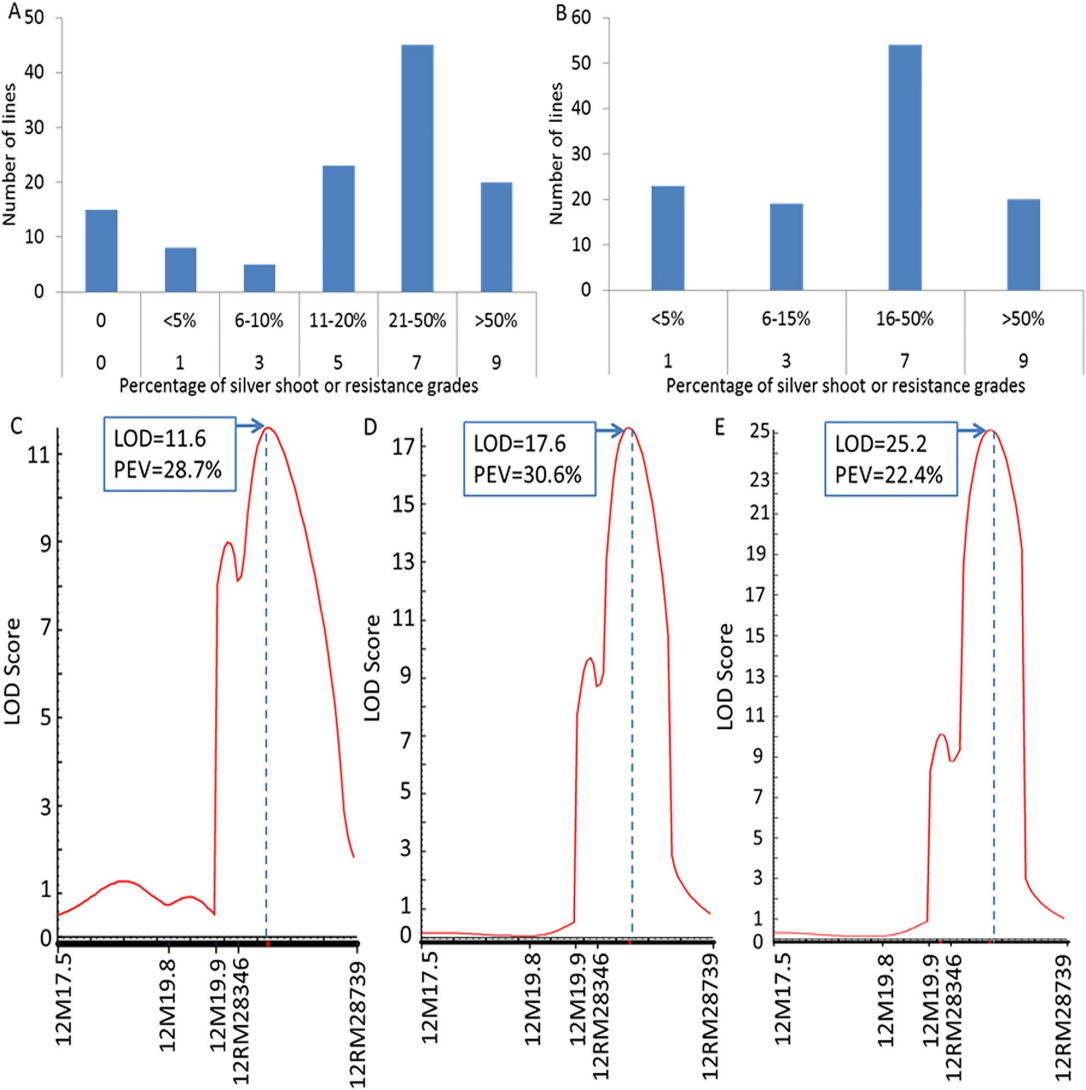
Rice gall midge (RGM) resistance gene mapping using conventional and improved grade systems. **A** Number of lines evaluated with the conventional grade; **B** number of lines evaluated with the improved grade; **C**–**E** quantitative trait locus (QTL) mapping with phenotypes based on percentage of silver shoots (PSS), conventional grade, or improved grade, respectively. Vertical dotted line indicates the location with the largest logarithm of odds (LOD) score. PEV, phenotypic variance explained by the locus

To verify the efficiency of the evaluation method with the simplified indicator, a novel F_3_ population (9311/CL6) was used to identify the resistance gene. The RGM resistance test was performed following method 2–2, i.e., treatment with 24 RGM, as described in Fig. 2B. Consequently, the PSS showed a continuous distribution and varied from 0 to 100% in the mapping population, and most of the lines were clustered at ranges of 11%–50% or 16%–50% according to conventional or simplified evaluation criteria, respectively (Fig. 4A, B).

To detect the linked markers associated with RGM resistance, a total of 1260 simple-sequence repeats and insertion-deletion markers were used to screen the extremely resistant and susceptible DNA pools. Subsequently, two polymorphic markers 12M19.9 and 12RM28346 on chromosome 12 were detected, and three other markers 12M17.5, 12M19.8, and 12RM28739 around the region of interest were identified as polymorphic between the two parents. All five markers were surveyed with the genotypes of the F_3_ population, based on which one local genetic linkage was developed (Fig. 4C–E). The QTL mapping results obtained using QTL IciMapping indicated a large locus between the markers 12RM28346 and 12RM28739 by analyzing the genotype and phenotype of the F_3_ population. Interestingly, the detected locus was identical when the phenotype was scored as PSS, conventional grade, or improved grade (Fig. 4C–E). However, the largest LOD score and phenotypic explained variance (PEV) value were different among them. Specifically, one locus with a large LOD score (11.6) and PEV (28.7%) was indicated when PSS was considered as phenotypic data, whereas when the conventional and improved grades were taken as phenotypic data, the LOD and PEV were 17.6 and 30.6% and 25.2 and 22.4%, respectively (Fig. 4C–E). These results suggest that all three indicators can be used as a phenotype to map the RGM resistance gene. It must be noted that the improved grade can simplify the evaluation method if the resistance grade is considered to be an indicator.

### Verification of the no-free-feeding method

The RGM without the free-feeding treatment is usually applied to check gene expression using qRT-PCR or to study transcriptomes and metabolomes. To verify the efficiency of the no-free-feeding test, one bucket with 24 seedlings was treated with six RGM adults infested for 0, 1, 3, and 4 days. The isolated RNA and cDNA were used for qRT-PCR analysis of the candidate genes [13]. The same treatments were applied to compare insect growth and development on the resistant line NIL-*Gm6* and susceptible line 9311 [9]. Moreover, identical experiments were performed to prepare the samples for transcriptome and metabolome analysis of NIL-*Gm6* and 9311. Specificly, ^1^H NMR spectra was applied to detect the metabolites of samples treated with RGM at 0, 5, 7, and 9 days. Consequently, more than 50 metabolites were identified including 4 kinds of sugars, 11 kinds of organic acids, 18 kinds of amino acids, 6 kinds of choline metabolites, 9 kinds of DNA/RNA associated nucleosides, and other kinds of metabolites such as ethanol and allantoin. In addition, transcriptomes of NIL-*Gm6* and 9311 were conducted at 0 and 7 days after RGM infested. The total number of induced different transcript genes of resistant and susceptible lines at 7 days was 585 and 1210 compared to untreated lines (0 day), respectively. Moreover, a total of 1111 and 678 genes were respectively suggested to be different in resistant line NIL-*Gm6* at 0 and 7 days comparing with those of the susceptible line 9311.

## Discussion

### Development and verification of an HTPM

Generally, rice insect pests can be classified as piercing sucking types, such as planthoppers, and chewing types, such as borers and RGM. Considerable progress in rice piercing sucking insect resistance, such as BPH resistance, has been achieved in the past decade [11]. In contrast, less progress has been achieved to date in host resistance gene identification and mechanism exploration for rice chewing pests. Exploring the likely reasons for this situation, an important restrictive factor has been the lack of an adequate phenotyping method that could be applied to evaluate the resistance level. Haghighattalab et al. [16] emphasized that high-throughput phenotyping platforms could provide the keys to connect the genotype to phenotype by increasing capacity and precision and reducing the time required for evaluating large germplasm populations. Both rice borers and RGM are chewing insects, and highly efficient phenotyping methods can greatly advance studies on them. However, rice borer resistance evaluation remains stagnant and no definite resistance line/variety has been identified to date. Fortunately, rice resistance to RGM has achieved some progress recently, and the resistance evaluation method should be taken into account [9, 13, 17, 18]. In the present study, 60 lines/varieties, including one or two control lines, infested with 36 female RGM could be treated in one tray as described by method 4–3 (Fig. 1), whereas only 12 lines/varieties could be surveyed using the conventional method. In addition, fewer insects were needed in the improved test, which could avoid the shortage of insects. This method is suitable for screening thousands of germplasms or mutants. For example, the ^60^Co-γ ray irradiation mutant screening test confirmed this (Fig. 2). Moreover, 14 lines/varieties with two replications could be evaluated in one tray as described by method 2–2 with 24 RGM used for infestation, which can increase the accuracy and efficiency of evaluation (Figs. 1 and 4). Taken together, an HTPM was developed successfully and applied efficiently to detect RGM resistance.

### Simplified evaluation method with improved indicators

Araus and Cairns [19] indicated that phenotyping of appropriate traits, using low-cost, easy-to-handle tools, should become an integral and key component in the breeding procedure. The improved evaluation method is simplified and easier to perform. Seedlings with inflated shoots or multiple tillers were considered susceptible according to the IRRI Standard Evaluation System and previous studies [6, 7]. However, insect development and growth were seriously restricted, and they finally died on the shoot according to previous observations, which caused invisible galls in certain lines [9, 13]. Rice plants are hypersensitive to RGM insects, which is called compatibility [4]. Therefore, these two types of seedlings were clustered as resistant when using the improved method. In addition, lines/varieties with PSS ≥ 16% were considered to be susceptible based on our previous observation, and the improved criterion was simplified to four grades in the practical RGM resistance test (Table 1). It was easier to evaluate the resistance level and avoid decision mistakes. No significant difference was observed when the conventional evaluation grade or improved grade was applied to detect RGM resistance gene mapping derived from CL6 (Fig. 4). The mapping results showed that the LOD score conferred by the resistance gene was higher with the improved criterion (25.2) than that of the conventional method (17.6) (Fig. 4). This suggests that the improved method could offer a more accurate analysis result.

### Increasing accuracy and precision of RGM resistance evaluation

HTPM and simplified indicators can be greatly beneficial to the RGM resistance test and contribute to its study. In addition, the accuracy and precision of the RGM resistance evaluation are also very important for the improved method. Twelve lines (containing two control lines) were evaluated using the method described by IRRI [6], whereas 15 lines/varieties with two replications could be evaluated in one tray using method 2–2 with 24 RGM treated for infestation (Fig. 1). Similar amounts of time and space required with more replications can increase the test accuracy. This method is suitable for hundreds of rice lines/varieties insect resistance tests and has been successfully applied to map RGM resistance gene resistance genes (Figs. 3 and 4) [9, 13]. Moreover, one cup/bucket with a fixed ratio of seedlings/insect treatment offered a novel method for the RGM resistance test, which can satisfy the requirements of insect development observation or molecular biological studies. Li et al. (2020) applied the method to detect the development of RGM on the resistant line NIL-*Gm6* and susceptible line 9311 [9], and Zhou et al. [13] conducted qRT-PCR to analyze the expression of candidate genes of *Gm5*. In addition, the developed treatment can survey physiological resistance mechanisms, such as antibiosis or antixenosis conferred by GM resistance genes [20, 21]. It can also meet the requirements of RGM insect genome or transcriptome studies, similar to those on other rice insects such as BPH [22]. Taken together, the developed method can increase the precision of RGM resistance and yield more accurate results in a practical study than the conventional method.

### Further considerations regarding the improved method

**Fig. 5:**
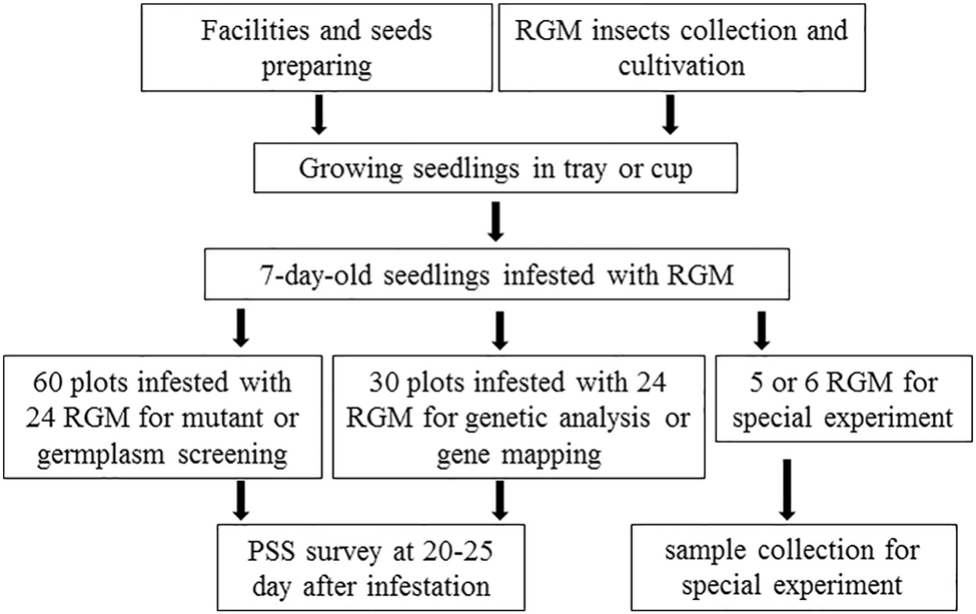
An overall flow chart of the experiment

The improved method is efficient, simplified, and precise. It can meet almost all RGM resistance tests requirements of modern breeding programs or molecular biological studies. Moreover, the implementation and observation of the test were easy for workers to perform according to the protocol (Fig. 5). Subsequently, the improved method was developed and performed under general conditions and does not require special equipment or techniques. Therefore, it can be easily applied to practical breeding programs. However, it should be emphasized that the improved method was only considered for evaluation of rice resistance to RGM at the seedling stage. Adult rice plant resistance against RGM is also very important [23], and it is possible that the same rice lines or varieties may show different resistance reactions to RGM at the adult stage. The same phenomenon was observed in a study on rice resistance to blast disease [24]. Therefore, further investigation is needed to explore the resistance of adult rice plants to RGM.

## Conclusion

An improved RGM resistance evaluation procedure was developed, and the PSS of rice seedlings was verified as a phenotypic indicator. Specifically, we presented an HTPM for detecting mutants and RGM resistance gene mapping using simplified phenotypic indicators. Moreover, we confirmed that the conventional method could be applied to conduct genetic analysis of RGM resistance genes using a mapping population. Finally, we developed a novel method to prepare samples for RGM insect growth and development observations and molecular biological studies. The improved or developed phenotyping procedure would significantly contribute to future studies and applications of RGM resistance.

## Data Availability

The data sets supporting the results of this article are included within the article.

## Abbreviations

RGM: Rice gall midge
IRRI: International Rice Research Institute
HTPM: High throughput phenotyping method
KW: Kangwenqingzhan
DAI: Days after infection
qRT-PCR: Quantitative reverse transcription-PCR
NIL: Near isogenic line
QTL: Quantitative trait locus
LOD: Logarithm of odds
